# Are Chinese Teams Like Western Teams? Indigenous Management Theory to Leapfrog Essentialist Team Myths

**DOI:** 10.3389/fpsyg.2020.01758

**Published:** 2020-08-11

**Authors:** Tomas Casas Klett, Jan Ketil Arnulf

**Affiliations:** ^1^University of St. Gallen, Research Institute for International Management, St. Gallen, Switzerland; ^2^Department of Leadership and Organizational Behaviour, BI Norwegian Business School, Oslo, Norway

**Keywords:** teams, China, leadership, essentialist theory, indigenous perspective, top management team (TMT), team process, team emergent states

## Abstract

Our study analyzes a gap in research on Chinese and Western management teams, based on a broad literature review. We claim that prevalent theoretical perspectives in the management team literature might be biased toward a Western-centric view of team dynamics. This obscures alternative ways of understanding top teams encompassing Chinese cultural traditions. We outline how an essentialist team conceptualization leads to a paradox consisting of three mutually contradicting myths. Myth 1 implies that Western groups of managers comply with theoretically “ideal” team processes and characteristics. Myth 2 derives from research literature on Chinese teams claiming that team features are assumed absent or weak in China due to cultural particularities. Paradoxically, the same research tradition constructs another third myth by reporting that Chinese teams successfully comply with the Western ideal team model. The three coexisting myths point to a theoretical confounding of contextual mediators in team processes. We discuss how indigenous Chinese leadership theory and Chinese systems of philosophy give Chinese teams access to distinct and effective team processes to reach high-performance outcomes. This paper aims to open the rich possibilities of Chinese management and team practices to the cross-cultural context, and on return to novel understanding of Western teams beyond traditional essentialist theory anchors.

## Teams: What we Know and Three Myths

Are successful management teams across the world successful in the same way? Or will culture shape how individual contributions are merged into performance? Reviewing the literature on management teams with an emphasis on cross-cultural research and in particular on China, we have identified a conceptual gap between theorized team features and the description of how culture influences the behaviors that lead to team outcomes and performance.

We believe that filling this gap will enhance the value of cross-cultural studies on management teams for practitioners. It will hopefully also open for the development of a truly geocentric understanding of management teams where knowledge from the East is adopted by researchers in the West. On the basis of a comprehensive literature study, we outline three “myths” about management. The paradoxes inherent in these myths serve as a conceptual frame to bridge observed differences in management team behaviors in the West and in China. The contribution of this piece, on one hand, is to increase our conceptual capability to incorporate cultural differences in research on management teams, and on the other, to stimulate empirical research that actually takes these differences into viable research designs.

The point of departure for our study is Hambrick's “upper echelon theory,” a research tradition focusing on the characteristics of top management teams in organizations (Hambrick et al., 1996; Hambrick, 2007). A central tenet in this theory sees diversity among top managers as an asset to the organization, a perspective that also seems valid in cross-cultural contexts (e.g., Li et al., 1999; Li, 2014; Chen L. et al., 2015; Li and Cui, 2018). However, there seems to be less research on *how* the team processes and social dynamics among such managers unfold their full potential, and whether these dynamics vary in significant ways across cultures. A common challenge in global leadership is for top decision-makers to achieve high performance where contexts call for different situational awareness (e.g., Russwurm et al., 2011; Gesteland, 2012; Gutierrez et al., 2012).

### Teams and Team Myths in China

An early study of TMTs in Joint Venture (JVC) companies in China, and building on upper echelon theory, was made in the early phases of Chinese economic expansion (Li et al., 1999). This study found, unsurprisingly, that the TMTs studied never qualified as “teams” in a theoretical sense. They were groups of managers struggling hard to overcome cultural barriers and their effectiveness was suboptimal to say the least. Interestingly, although the article foresaw two decades of cross-cultural leadership trouble in JVCs (e.g., Wong et al., 2018), it never made any references to Chinese culture and philosophy as explanations for phenomena or suggestions for solutions. The analysis and suggestions from 1999 were made entirely from Western team theory perspectives. This is a clear example of what Li (2012, p. 852) describes as naïve exploitative Western research in Chinese management, the first of four phases of research development toward truly indigenous, geocentric contributions. The first phase is characterized by pure exploitation, an uncritical local application of pre-existing Western theories, which Li calls “emic-as-etic.” This is the belief that what works in the West universally works everywhere. In the second stage, the interest turns toward a comparative research approach spanning multiple contexts called “etic-to-emic”—opening up for local variants. At stage 3, the research will recognize and try to explain a uniquely local phenomenon. This “emic-as-emic” approach will recognize uniqueness. Finally, only at stage 4 will research acknowledge that discovery goes both ways—what was found to be uniquely local will also be found to inform the original general research tradition. A true geocentric approach is where Chinese approaches to teams and leadership phenomena are accepted on their own accord with consequences for research in the West and elsewhere.

While the researchers involved in the 1999 study cited above went on to work on contextualizing Chinese leadership research, our impression from reviewing even recent literature on TMTs in China is that the status of research has not reached the final stage of Li's model. We want to show that research on management teams in China and the West may have overlooked the powerful contribution of how Chinese culture and thought systems shape the minds of Chinese managers and guide their interaction. An overarching research question therefore bears on our analysis: Is research on Chinese top management teams (TMTs) stuck in the emic-as-etic perspectives where Western theories are treated as essentialist descriptions of team dynamics?

From the literature we derive three mutually contradictory myths, the first of which is labeled “Ideal Team (Myth 1)”: Are there ideal team characteristics that are necessary prerequisites for successful performance of groups of top managers? Or are these characteristics neither necessary nor representative, but rather a rare or even a mythical state for Western teams? The second question is labeled “Chinese Non-Team (Myth 2)”: Do Chinese groups of managers display different characteristics from Western teams due to cultural traditions for communication or power distance, thus compromising the high-performance potential of team dynamics? The third question is labeled, “Chinese Teams Like Western Teams (Myth 3)”: Do research findings report that team characteristics and emergent states of management teams in China do not set them apart from Western teams, implying that Chinese cultural differences do not matter?

Li (2012, p. 851) suggests that indigenous research is characterized along four core dimensions: “What,” “Why,” “How,” and “For Whom.” Applying this framework to our study, we get the following perspectives:

#### “What”

The target of our study are possible models of team behaviors and interactions that are easier to access and develop in China than in the West, due to Chinese culture and philosophical traditions. We will focus on two conceptual features of team theory where the second seems strongly associated to traditional Western tenets: (i) a proper “team” is not a mere collection of members in a group, but a dynamic structure with special characteristics and discrete emergent properties. This seems like a generic description that separates a “team” from a mere “group.” (ii) The second feature however, especially in its normative variant, is heavily dependent on horizontal communication and (relatively) autonomous self-organization, making team functioning antithetical to centralized hierarchical control. As we will show below, this feature of teams is usually referred to in research as culturally sensitive. The origin of the word “team” is also a metaphor, and it has therefore been conceptualized in various ways over the years (Cohen and Bailey, 1997; West et al., 1998; Hackman and Wageman, 2005). As we argue below, this imprecision has complicated the theory linkage to empirical evidence of how team processes drive performance. A conceptual barrier has disallowed unequivocal exploration of teams across cultures.

Questions and findings pertaining to research on teams in general, and top management teams (TMTs) in particular, will be strongly dependent on how strictly the researchers define and operationalize these terms. It is our claim that ethnocentric definitions, theories, and operationalization of teams inhibit effective cross-cultural research on teams as explanatory variable for organizational performance. While no one disputes that most modern companies are headed by groups of managers, the nature of their co-operation, and social dynamics can be expected to vary across cultural contexts (Li and Cui, 2018; Zhang et al., 2018).

#### “Why”

Through reading the cross-cultural research literature on management teams with focus on East Asia, we believe to have uncovered three mutually contradicting myths about top management teams, and articulate the myths as one paradoxical structure in the discussion of East-West distinctions. These myths are theoretically interesting, as they point to contradictions and enable us to address gaps in the literature. The myths are central to theory and to the subsequent operationalization of team research, as well as to the intuitive ideas about managers in teams.

#### “How”

After identifying the three myths and the resulting gap, we will review research and outline how Chinese philosophy can help advancement of alternative models of management team dynamics. Our study connects team functioning and their cultural mediators (in the sense of Ilgen et al., 2005) by differentiating between the processes that characterize high-performing groups of managers and the culture elements and thought systems that impact and mediate team processes. We will build on an emerging research tradition of indigenous Chinese theories of leadership to describe some types of management teams as a “imperial court” as different from what is usually meant by the term “team.”

#### “For Whom”

First and foremost, we will argue in line with Li (2012) that truly indigenous, “Eastern-as-emic” studies are enriching to a truly geocentric body of research. Our position is that Western theories will profit from adopting indigenous perspectives from other parts of the world. Concomitantly we also think that practitioners benefit from prescriptive implications more adapted to local context.

In what follows, we will apply these four dimensions to explore our research questions.

### Myths as Knowledge Gaps in Team Theory

Theoretically, teams differ from mere groups on account of their emergent properties and states. These cannot be reduced to the individual contributions of each participant (Wilson, 2001; van Knippenberg and Mell, 2016). While the word “team” has been assigned to all sorts of tasks carried out by groups of people, the idea of a “management team” necessarily carries special meaning. Many technical tasks may be carried out as routine instrumental interactions with little verbal exchange, but management is essentially about communication, problem solving, and other intellectual tasks (March and Simon, 1958; Day et al., 2004, 2006; Khurana, 2007; Mintzberg, 2009; Wang et al., 2014). Teams in management have therefore been of special interest to management research for the reason that they necessarily contrast the idea of centralized decision making with a distributed, decentralized approach (Belbin, 1981; West et al., 1998; Hackman and Wageman, 2005; Hambrick, 2007; Carmeli et al., 2012; de Wit et al., 2012).

Since the 1990s, management teams have been presented as a key to organizational adaptation, and potentially conducive to top performance. First, the top-echelons perspective describes how TMT member characteristics such as education, experience, and demographics are especially valuable assets to the organization (Hambrick, 2007; Mackey, 2008; Hansen et al., 2013). Second, team theory suggests that the dynamics, and synergies in diversity between the members are an important condition for the successful utilization of the various assets the management team *a priori* has at its disposal (West, 1996; Beer and Eisenstadt, 1999; Jehn et al., 1999; Hackman, 2002). The theoretical problems of taking an essentialist perspective and obviating indigenous insight in team research in cross-cultural contexts will become evident as we review the literature behind the three myths. Normative cross-cultural perspectives of leadership ought to incorporate local cultural elements and systems of thought such as traditional Chinese philosophies (Lin et al., 2018, p. 301).

#### Myth 1 “Ideal Team”

First, the well-documented “Ideal Team (Myth 1)” disputes that groups of top managers are actually teams in the true, theoretical sense of the word. The identification of this myth goes back to two influential articles from *Harvard Business Review* over two decades ago. The first promoted the idea of diversity and task conflict in “How Top Management Teams Can Have a Good Fight” (Eisenhardt et al., 1997). The second article contested the very idea of “teamness” or horizontal, shared, collaborative leadership in groups of top managers as the “Myth of the Top Management Team” (Katzenbach, 1997). Katzenbach argued that “teamness” and the emergent properties that make teams distinct from mere groups are rarely on display in TMTs. Hambrick, one of the main proponents of top echelon theory (Hambrick, 2007, p. 336) underscores this myth asserting that “members of TMTs consist of semiautonomous ‘barons,’ each engaging in bilateral relations with the CEO.” The problem of unifying diverse perspectives has become a core focus in on-going controversies in team theory, for instance in the discussion about how cognitive task conflicts also turn into emotionally disturbing relationship conflicts (De Dreu and Weingart, 2003; van Knippenberg et al., 2004). The debate in team research continues (de Wit et al., 2012). In other words, it is unsettled whether team characteristics really apply to management teams in general, and to groups of top managers in particular. The “top” of the term “top management team” refers to the organizational level, not to ideal horizontal leadership or “teamness,” and is therefore a misnomer in a strict team theoretical sense—its prevalence points to sloppy theoretical compliance.

If TMTs are a possible myth, how likely is it then that company performance is a function of TMT composition and processes? The characteristics of top echelons teams have been shown to influence teams through broad and diverse composition. To make diversity useful, team processes must somehow integrate diverse characteristics in the management of the company (Lewis et al., 2005, 2007; Hambrick, 2007; Cannella et al., 2008; Bjornali et al., 2016; Li and Cui, 2018). Integration effects have been found to occur in different countries. But does this mean that team leadership and “teamness”—or lack thereof—will be the same across all cultures?

#### Myth 2: “Chinese Non-Team”

As a kind of mirror image of the “Ideal Team (Myth 1),” which is centered on Western team research, there has been a *tacit* myth that Chinese top teams do not exist. This is the “Chinese Non-team (Myth 2).” Unlike the explicit and ample literature directly conceptualizing “Ideal Team (Myth 1),” this second myth is documented through our broad review of the research literature on Chinese teams.

When researchers study phenomena such as voicing opinions, task conflict, and distributed leadership in a Chinese context, they frequently argue that their studies are interesting *precisely* because team processes are assumed to be less likely to occur in China than elsewhere (e.g., Chen and Tjosvold, 2005, 2013; Chen Y. F. et al., 2005; Fu et al., 2008; Wang et al., 2010; Ou et al., 2014; Tjosvold et al., 2014; Li and Cui, 2018). The “Chinese Non-Team (Myth 2)” is thus implicit in the theoretical positioning of an expanding literature on Chinese teams. Outside the realm of academia, this myth is complemented by anecdotal evidence, sometimes articulated by lay Chinese people when they discuss their football team's (under-)performance, or in sayings like, “One Chinese is a dragon, one [chose a foreign nationality] is an ant; a hundred Chinese are a hundred ants, a hundred [foreigners] are one dragon.” To conclude, the “Chinese Non-Team (Myth 2)” appears to be a recurring assumption in research of many Chinese and Western academics, and is at times even articulated at lay levels in China. This of course is in direct conflict with evidence on high-performing Chinese teams, including examples that this paper reviews. One has to look no further than to Mr. Jack Ma's organizational changes which, in his own words, include breaking Alibaba up “into ‘more’ small business operations” in order “to give more young Ali leaders the opportunity to innovate and develop” (Ma, 2013). The focus is on each “business and their team”—Alibaba might ultimately be an organization of teams.

#### Myth 3: “Chinese Teams Like Western Teams”

We now turn to a third myth. Its critical analysis is a main topic of this study and frames our contribution. The core of “Chinese Teams Like Western Teams (Myth 3)” is that management teams actually do exist in China *in the same manner* as in the West because the same team functions are found in Chinese team research. These academic findings are regularly presented as proving the existence of Western ideal team functioning, states and processes where there were reasons to expect their absence in accordance with the “Chinese Non-Team (Myth 2).” In other words, the published research assumes that the same systems of thought and social mechanisms that apply in Western teams also operate in China. The implication is that indigenous Chinese mechanisms might not be noteworthy and can safely be ignored. In other words, the essentialist premise sees teams and their processes as universal, the same everywhere.

The empirical support for Myth 3 falls broadly into three categories: The first type of studies applies the concepts from management team theories such as “Top Echelon Theory” (e.g., Hambrick, 2007) to explore archival data from listed companies. One such example is Li and Cui (2018), exploring the effect of TMT functional diversity on ambidextrous foreign investments and arguing that task conflict is the mediating mechanism. However, the effect sizes are small (around 0.10) and the team mechanism are only inferred, not studied directly. The second type of studies are conversely studying team mechanisms directly but usually only access lower level management teams using Likert-scale surveys, frequently with cross-sectional self-reported data. One example of this is a study by Zhou et al. (2015) that looked at the role of personality composition in entrepreneurial teams. The effect sizes they found ranged from small to medium-sized (0.16–0.31) and even though the study was conducted in Eastern China, there was no discussion of how culture may have played a role in mediating the effect of team personality composition. There is an over-reliance on cross-sectional self-report data prone to inflated statistics due to common method variance (Podsakoff et al., 2003). The data structures in cross-sectional Likert-scale data are often driven by semantics that are insensitive to cultural differences and explain up toward 80% of the variance (Arnulf et al., 2014). A recent study using semantic methods found that frequently used measurement instruments in leadership surveys are not capable of detecting cross-cultural differences (Arnulf and Larsen, 2020). On this basis falsifiable hypothesis leveraging the semantic research paradigm might empirically test whether cross-cultural team research is asserting universality of team constructs on account of semantic structures, and in that case possibly understate the true impact of cultural differences on team behaviors.

We have found only very few studies of the third category that combine data on the actual mechanisms within the teams and non-survey data on firm performance. One such exemplary study was carried out by Li et al. (2016) on the effect of open debate in management teams and the effect on the company's performance, measured by Tobin's Q and mediated through ambidexterity. In this study, the effects, first of ambidexterity on Tobin's Q and of open debate on ambidexterity, were medium strong (0.27 and 0.37, respectively). We believe that there is room for methodologically stronger studies that take indigenous theories of management team dynamics into consideration, in the way that leadership research in China has improved through the application of theoretical frameworks like paternalistic and paradoxical leadership behaviors (e.g., Farh and Cheng, 2000; Ma and Tsui, 2015).

The three myths are succinctly exposed below in Table 1, together with their sources and the position on their existence that is taken in this paper.

**Table 1:** Summary of three team myths, including two Chinese team myths.

	**Phrase**	**Description**	**References**	**Position**
Team Myth 1	“Ideal Team”	There are no true teams; ideal teams with properties and states as normatively proposed in the literature rarely exist at top management teams (TMT)	Belbin, 1981; Katzenbach, 1997; Naquin and Tynan, 2003; Hambrick, 2007	Myth 1, partially accept Ideal “teamness,” horizontal leadership, is a matter of degree; both ideal and non-ideal teams exist on a continuum
Team Myth 2	“Chinese Non-Team”	China has no real or ideal teams, and thus low performance. The assumption is that ideal team processes are less likely to occur in China. Chinese favor hierarchies, and are shackled by Confucian heritage. This is supported by lay sayings and urban legends like Chinese football teams' “perennial” (under-)performance	Chen and Tjosvold, 2005, 2013; Chen Y. F. et al., 2005; Fu et al., 2008; Wang et al., 2010; Ou et al., 2014; Tjosvold et al., 2014; Li and Cui, 2018	Myth 2, reject Chinese high-performing teams exist, and they can display ideal team states and processes. However, these are not explained by essentialist team theory. The claim is that indigenous Chinese culture and thought systems do not prevent high-performance; rather they explain it, as they explain Chinese team processes and states
Team Myth 3	“Chinese Teams Are Western”	Chinese teams are akin to Western teams. Myth 3 would be an anti-myth, which contradicts both Myth 1 and Myth 2	Zheng, 2012; Cai et al., 2013; Li, 2014; Dai et al., 2016	Myth 3, reject Chinese teams perform (or non-perform) partially, and not in decisive fashion, on the basis of essentialist Western team theory. Indigenous Chinese culture and thought systems mediate Chinese team processes, states, and performance

**Figure 1:**
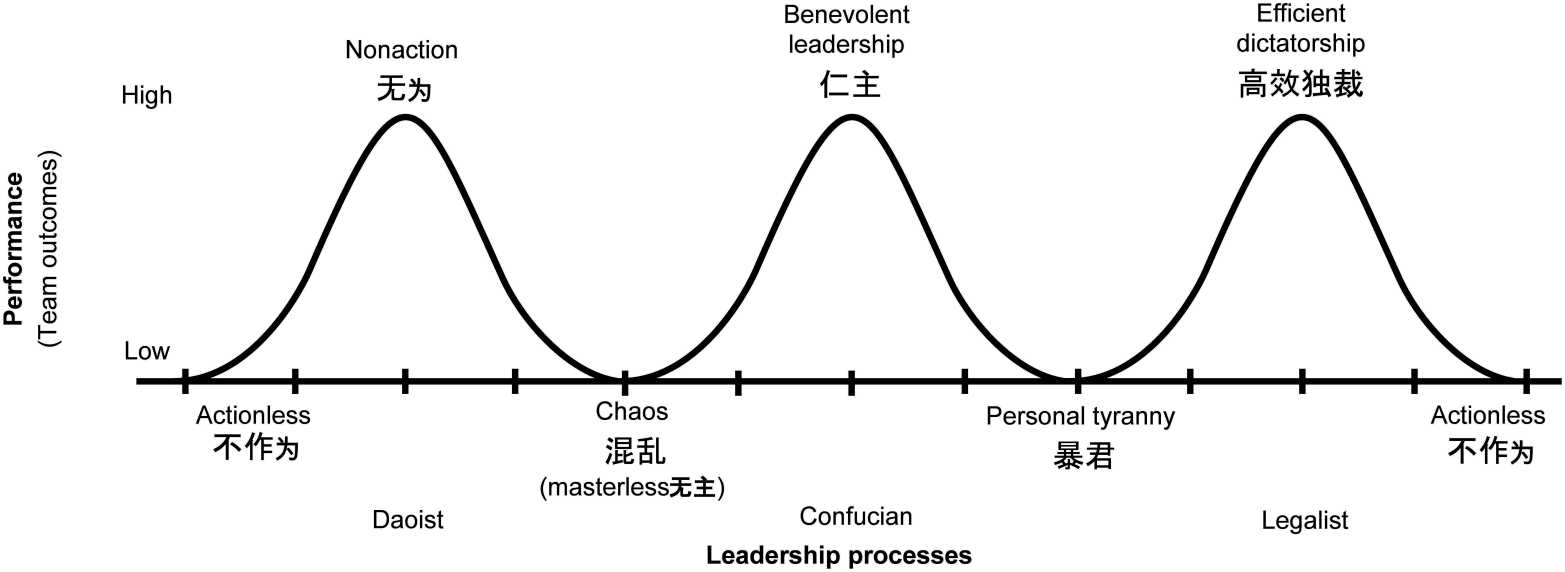
Chinese thought systems mediating leadership styles leading to team outcomes.

We now turn to the implications of the three myths on theory development. While leadership processes of managers should bring about true team dynamics, practice shows that this is often not the case. TMT research thus suggests a fallacy, the “Ideal Team (Myth 1).” The expectation that indigenous Chinese leadership practices prevent open flow of communication and shared decision making, “Chinese Non-Team (Myth 2)” has only a basis in radical essentialist theory. The question then has to be whether it makes sense to assume that team dynamics in Chinese management teams are really the same as in Western management teams—the “Chinese Teams Like Western Teams (Myth 3).” Or would it not be more reasonable to theorize that the different thought systems, cultural and social dynamics of East and West lead *ceteris paribus* to similar performance albeit in different ways? The rest of our study answers the question positively and argues against the assumption that teams across cultures are essentially identical. The theoretical development associated with this position becomes specific in the next section with a proposed conceptualization of how diverse Chinese thought systems mediate and become an explanatory variable for leadership styles and so lead to specific team outcomes (see Figure 1).

### Anchored in the Indigenous Approach

If emergent states in social groups are context—and hence culture-dependent—then applying straight Western management theories in China would be futile or even counterproductive (Lin and Su, 2014). Emergent states which “describe cognitive, motivational, and affective states of teams, as opposed to the nature of their member interaction” are “constructs that characterize properties of the team that are typically dynamic in nature and vary as a function of team context, inputs, processes, and outcomes” (Marks et al., 2001, p. 360; Ilgen et al., 2005). Are culture and the associated thought systems critical team context? And, if so, what are the theoretical implications?

The problem can be validly examined by looking at groups of top managers through cultural lenses. A review of Chinese management studies (Zhang et al., 2014) delineates three distinct ways of doing such research: as essentialist and context-free replications in China (equivalent to Li, 2012 stage 1), as adaptations to China of Western research (Li's stage 2), or as truly indigenous approaches conceptualized and documented from a Chinese perspective (Li's stage 3). This paper leverages the indigenous approach to take a fresh look at “Chinese Non-Team (Myth 2) and its anti-myth, “Chinese Teams Like Western Teams (Myth 3).” Chinese firms are becoming increasingly global and innovative, creating a need for essentialist management research to aim at pluralistic insight and deep contextualization (Tsui, 2006; Tsui et al., 2007), establishing a geocentric perspective in the sense of Li's stage 4 (Li, 2012). It is the right time to take a fresh look at both Chinese team myths. Yet before the theoretical discussion indigenously addresses Chinese teams, we must provide a critical review of the essentialist theory of management teams.

## General Theoretical Overview: Teams vs. Organizations

The literature distinguishes teams from mere groups on account of the members' complementary roles, interdependence of tasks, shared responsibility for outcomes, and perceived identity across organizational boundaries (e.g., Cohen and Bailey, 1997; West et al., 1998; Jehn et al., 1999; Hackman, 2002; Day et al., 2004; van Knippenberg and Mell, 2016). To qualify as a team, there must be some degree of self-governing adaptation or the presence of certain team processes such as “shared leadership” (Ensley et al., 2006; Wang et al., 2014). There seems to be agreement that a group of specialists that are organized and led hierarchically is not a team. This assumption starts the theoretical journey into the essentialist mainstream conceptualizations of teams.

### Mainstream Team Theory

Empirical studies keep finding that the scope of resources available to the management team exceeds the resources of the CEO alone, and hence the expected positive impact of management teams on innovation, strategy, decisions, and execution (e.g., Hambrick et al., 1996; Edmondson et al., 2003; Carmeli and Schaubroeck, 2006; Cannella et al., 2008). Team theories explain how complementary resources are brought proactively into play by each member and contribute to, for instance, the TMT's cognitive emergent states, such as shared mental models (SMMs) or transactive memory systems (TMS) (Lewis et al., 2005; Dionne et al., 2010). Conversely, it has consistently over the decades been assumed that forces opposing the emergence of team qualities will restrict the performance of teams, including top management teams (Steiner, 1972; Hackman, 1987; Frey, 1996; West et al., 1998; Homan et al., 2007; de Wit et al., 2012).

The role of culture or context in teams has not been extensively developed within the framework of team theory. Early theories took a mechanistic view that emphasized an essentialist perspective. McGrath's (1964) input-process-output (IPO) model provided a framework for team functioning under the assumption that members use their complementary resources to monitor and proactively contribute in team processes. Optimally, the contribution by each member is absorbed to the benefit of the team, albeit subject to process loss as the cost of coordination (Steiner, 1972). While team models purport no normative “teamness” gold standard (Hackman and Wageman, 2005), there is agreement that affective emergent states such as trust and the psychological safety of team members also play an important role in stimulating proactive or self-adjusting behaviors (Edmondson, 1999; Burke et al., 2006; Carmeli et al., 2012; Koopmann et al., 2016).

More recent adaptations took a more dynamic view of team phenomena, and opened the door to the role of culture. According to the IPO derived input-mediator-output-input (IMOI) model of Ilgen et al. (2005), emergent states which “describe cognitive, motivational, and affective states of teams, as opposed to the nature of their member interaction” are “constructs that characterize properties of the team that are typically dynamic in nature and vary as a function of team context, inputs, processes, and outcomes” (Marks et al., 2001, p. 360; Ilgen et al., 2005). Culture can be seen as team context and this assumption enables the claim of Chinese thought systems as meditators of leadership styles (Figure 1). IMOI thus opens up the possibility of mediators of a cultural nature: “Substituting ‘M’ for ‘P’ reflects the broader range of variables that are important mediational influences with explanatory power for explaining variability in team performance and viability” (Ilgen et al., 2005).

Despite the modern emphasis on emergent properties, a “non-team” is decidedly a group whose function is restricted by a team leader who controls team processes. There is a potential theoretical link here to indigenous perspectives, which interestingly is never made explicit or articulated, because the non-team conceptualization fits exactly with how traditional paternalistic authority is conceived as functioning in China (Farh and Cheng, 2000; Cheng et al., 2014). This is where the “Chinese Non-Team (Myth 2)” originates. A great number of studies on Chinese team behaviors start by arguing that they study team behaviors such as voice, task conflict, and shared leadership precisely because these are commonly assumed to be absent (e.g., Tsui et al., 2003; Chen G. Q. et al., 2005; Fu et al., 2008; Zhang et al., 2011; Chen and Tjosvold, 2013; Li and Cui, 2018).

Our review of these studies shows that they generally end up finding that team properties are present in China, contrary to initial expectations. What started as tacit but misguided indigenous theorizing ends up as essentialism. While not explicitly claiming that culture is unimportant, this line of research fuels the essentialist view that team phenomena are similar everywhere, thus leading to the “Chinese Teams Like Western Teams (Myth 3)” posited earlier. More specifically, this research has shown how diverse and complementary management team resources are linked to ambidexterity and better firm performance (Fu et al., 2008; Liu et al., 2009; Li et al., 2016; Li and Cui, 2018), or that innovation is spurred by cognitive task conflict (He et al., 2014), and such conflicts can be handled at an optimal level so as not to turn into counterproductive relationship conflicts (Farh et al., 2010). Just as in all other contexts, psychological safety and trust mediate team performance through voice (Wang et al., 2010) and empowerment (Chen and Tjosvold, 2012; Hempel et al., 2012; Chen S. et al., 2015; Dai et al., 2016), in such a way that knowledge is shared and not concealed (Ma et al., 2014; Huo et al., 2016).

In summary, much research of Chinese teams underlines two irreconcilable yet complementary myths, which pose a theoretical and empirical paradox that needs addressing. Essentialist approaches start and justify the theoretical inquiry of management teams from a perspective of difference, but end up finding similarity. Ironically, the “Chinese Teams Like Western Teams (Myth 3)” has as necessary prerequisite, the “Chinese Non-Team (Myth 2).”

### Three Myths Imply a Research Agenda

The proposed three-myth structure is no rhetorical strawman because it yields three different interpretations of existing research that are non-trivial from theoretical and practitioner perspectives alike. The first is that despite being frequently assumed, there are no differences between Chinese and Western teams of managers. However, the “Chinese Teams Like Western Teams (Myth 3),” where *Chinese teams are really like Western teams* not only seems contrary to common sense, it clashes with a body of research in fields like international cooperation, where the differences cannot be ignored (e.g., Li et al., 2002; Selmer, 2005; Chrobot-Mason et al., 2007; Li and Li, 2009; Arnulf and Kristoffersen, 2014; Raghuram and Fang, 2014).

The second interpretation implies that *Chinese teams have become or are becoming Western teams*. This would argue that differences are disappearing due to economic development, industrial transformation, and institutional changes in China. This is equally unlikely, first because the cited team research spans 15–20 years. Moreover, the vast size of the Chinese society and the geographical spread of sampled teams would make a sweeping homogenous social transformation difficult. Lastly, if such a transformation were occurring, it would surely not be in the direction of convergence with the West, as the by-now obsolete cultural convergence theory would posit (Kerr, 2013).

We are then left with a third interpretation, that *the impact of context is obviated or misinterpreted in both Western and Chinese teams*. This might be the most interesting theoretical explanation for the three-myth structure paradox. Team research addresses complex social phenomena, and with the traditional focus on *team inputs, processes, and emergent states, it appears that the mediating effects of context and culture on emerging states constitute a research gap*.

The “Ideal Team (Myth 1)” suggests that Western teams are mythical. This seems partially corroborated through research and practice, especially when organizations perform well without necessarily relying on “teamness,” horizontal leadership, and “ideal” properties or team states (or even without relying on teams at all). Critics of the team as an organizational centrality have noted its fragile link to organizational performance. For example, even ideal teams sometimes fail (Naquin and Tynan, 2003; Mathieu et al., 2008; Wang et al., 2014), and few would conceptualize Steve Jobs, Donald Trump, or Larry Ellison as consummate team players, despite their apparently significant successes as business leaders. Team organization is also perceived to entail too much process loss, as in the saying that “a camel is a horse designed by a committee” (Frey, 1996; Taylor and Greve, 2006; Antoni and Hertel, 2009; de Wit et al., 2012). In addition, TMTs may take very different forms (in terms of processes and emerging states), as in Silicon Valley ventures or on a DJIA board. In short, the same desirable outputs can emerge in ideal teams as well as in hierarchical non-teams. Given this state of knowledge, an interesting perspective emerges through the assumption that different cultural contexts lead to both dissimilar and similar processes and emerging states which, in turn, lead in distinct ways to both similar and dissimilar team outcomes like high-performance.

What does eliminating the demand that all teams comply with essentialist Western perspectives entail? A recent stream of mostly Chinese management scholars, reclaiming Chinese cultural roots, make an important theoretical contribution by reinterpreting how organizations can be more than the sum of their parts—the individual members—in terms of organizational performance (Chen and Lee, 2008; Ning et al., 2012; Zhang et al., 2014; Arvey et al., 2015; Ma and Tsui, 2015). The argument raised by such researchers is that different cultural traditions open access to different follower behaviors. The rich philosophical and cultural traditions of China allow companies to display team processes like those theorized by existentialist theory, and reach high-performance team outcomes, but by means of indigenous forms of interaction, IMOI-mediating variables, and distinct emerging states. These can include affective (e.g., trust) and cognitive states (e.g., SMMs) that are activated by the participants' cultural context. The implications are profound, as team theory can thus innovate through indigenous conceptualizations.

Specifically, recent contributions to Chinese leadership theory argue that Chinese leaders and group members are able to embrace paradox, allowing different modes of authority, and cooperation to exist side by side (Chan et al., 2013). Further, they posit that authoritarian leadership in China is not monolithic, but combines the directive momentum of Legalist leadership with benevolent, trust-inducing aspects (Cheng and Wang, 2015; Chen L. et al., 2015; Zhang et al., 2015a). As research documenting this culturally dependent leader-follower dynamic accumulates, it is reasonable to theorize about the effect on team dynamics as well. For example, how may affective emergent states resulting from authoritarian leadership elicit the cooperative capabilities of members and a collectivist community (Wang and Young, 2013; Li et al., 2014; Ma et al., 2014)?

### When “Team” Is Both a Scientific Concept and a Cultural Metaphor

Originating etymologically from the ancient Germanic word “zaum,” the original meaning of the English word “team” was a collective measurement word for ox—a “team of oxen”^1^. It is also etymologically related to “taming,” as the “zaum” or bridle would render the wild beasts “tame,” i.e., docile and receptive to command. Through sports and other social settings, the word ended up as a descriptive of human collectives with dynamic properties. The word “team” is itself a metaphor.

When using “team,” theorists need to be precise given the possibilities of the term. First, “team” is a colloquial word that can be used interchangeably with “group” (all groups of managers are then also “teams” with no special requirements). Not distinguishing between teams and groups would go counter to research aiming to identify the discrete properties of high-performing groups. Second, “team” may be seen as an essentialist form of social organization, where not all groups are teams, but all ideal teams share common “teamness” properties, processes and states. Third, there is the notion that “team” describes social dynamics at small groups. Teams may then take a diversity of shapes in terms of their properties, processes and states, some of which are decidedly different from the essentialist “team.”

The perspective of this paper is the latter. The claim has been made that the essentialist idea of “teams” has blocked our understanding of group dynamics, especially when these are mediated by culture. As an alternative to ideal “teams” and to shed theoretical light on Chinese teams, we propose to employ the culturally-loaded metaphor of an “imperial court” as the entourage of the imperial “emperor” figure. This metaphor deliberately indicates a distinct social dynamic, consistent and associated with hierarchical structures. But does this metaphor invariably point to indigenous Chinese forms of leadership irreconcilable with, let's say, non-hierarchical team processes? Moreover, could “imperial court” processes lead to high-performance, or to high-performance akin to ideal Western teams?

In what follows, we build on Chinese philosophy and systems of thought, as well as on empirical case evidence, to conceptualize a dynamic high-performance “imperial court,” which would stand in conceptual contrast to ideal, essentialist Western top performing teams. Its team properties, processes and emergent states must however not be opposite to essentialist counterparts across the board. The conceptualization of Chinese leadership styles mediating team outcomes (Figure 1) is the critical analytical element, as well as the theoretical basis for alternatives to both “Chinese Non-Team (Myth 2)” and “Chinese Teams Like Western Teams (Myth 3).”

## Thought Systems as Mediators for Chinese Team Processes

Ancient systems of thought still exert powerful influence on thinking, learning, and management in China. Unlike Western traditions and narratives, Chinese philosophy is rarely taught through long texts (although these exist) or sacred books, but is expressed as aphorisms that often live on as proverbs that most people know (Mou, 2009; Feng, 2015). These powerful traditions form a most important background to understand the emerging theories of Chinese management (Zhang et al., 2014; Ma and Tsui, 2015), as Chinese have a tendency to leap from philosophy to pragmatic action, bypassing theory (Ralston et al., 1999). Chinese classics have been shown to be important strategic guidelines for Chinese business leaders (Chen and Lee, 2008). Peng and Nisbett (1999), Nisbett et al. (2001), Norenzayan et al. (2002), Nisbett (2003), and Ji et al. (2004) believe that philosophical traditions are not imposed on people by philosophers, but rather are explications of the world to the participants of specific social systems. Different traditions of work and subsistence in East and West have given rise to different modes of cognition that affect communication and coordination (Snell, 1982; Fei et al., 1992; Gumperz, 1996). Chinese thought systems have been conceptualized in “context-sensitive” research (e.g., economic reform) as context acting “as either main effects or as moderators” which extend and modify theory (Tsui, 2004), while at team models with emergent states of a psychological nature (the afore-reviewed IMOI) these would function as cultural mediators. We therefore make a brief review of team phenomena in light of its manifestations in Chinese thought systems.

### Sources of Chinese Thought Behind Three Indigenous Leadership Styles

Organizations everywhere struggle to balance centralized control with optimal levels of autonomy and laissez-faire, paralleling what team literature describes at times as hierarchical and shared leadership approaches. China's philosophical schools can be seen to systematize these models in the ways they conceive authority. A simplified presentation of the three principal schools of Chinese thought—Confucianism, Legalism, and Daoism—may capture the key context variables that mediate at Chinese team processes and emerging states.

Confucius or Kong Zi (孔子), a pre-imperial era scholar and adviser to the nobility in Shandong, saw tradition and authority as essential for order, which begins with the family and ends at the state (Confucius, 2009). This is the thought system dimension behind the “high power distance” attributed to China in management theory (Hofstede et al., 2010; Hackett and Wang, 2012). Filial piety is demanded from the subordinate, whose respect and deference to the leader is reciprocated from the top by benevolence. Confucian tradition emphasizes the marriage of authority and benevolence, a leadership style called “paternalistic leadership” (Farh and Cheng, 2000; Cheng et al., 2004; Farh et al., 2010). A recent example is Mr. Zhongqun Mao, the founder of high-end kitchenware manufacturer FOTILE. Not only does he lecture on Confucianism, encourages employees to read the Master, and has even established a “Confucius Hall”; he also “walks the talk” by letting the firm paternalistically “take care of its employees” by, for instance, investing in cultural and recreational facilities. However, decisions are made at the apex, by the owner family and when there is a task conflict (relationship conflict would be unlikely) at that top team level it is mainly between father and son—then Mr. Mao the elder might benevolently defer to his son, even when he disagrees (Liu and Heler, 2012).

The easily observable Confucian hierarchies and derived behavior, even if benevolent, are probably the main argument behind the “Chinese Non-Team (Myth 2).” While Western business structures in China have been visibly and successfully introduced in the Confucian context for more than 100 years (Chan, 1996), open controversy in the presence of a CEO, for instance, is not seen by many as a natural part of Chinese management (Peng et al., 2008; Confucius, 2009). Confucian tradition is seemingly anathema to the norms of Western teams, which assume benefits from task and process conflicts (Jehn et al., 1999). In the West, this conflict perspective was celebrated in the previously mentioned *How Management Teams Can Have a Good Fight* (Eisenhardt et al., 1997). In short, and notwithstanding attempts to transplant Western structures to China, especially in the early stages of multinational business entering the market (Li et al., 1999), indigenous leadership approaches seem to have prevailed.

Foreigners seeing a Chinese *laoban* (老板) or boss, managing his top team as an “imperial court” might quickly assume that Confucian hierarchies cannot have it any differently, and that leadership can only be vertical. Chinese CEOs are famous for their solitary splendor and might (Pi and Lowe, 2009). Mr. Zhang Yue, CEO of Broad Group, who aims to own 30% of the global construction industry with a “revolutionary but largely unproven technology,” is an illustrative case (Anderlini, 2016):

All Broad Group job applicants must undergo a week-long military-style boot camp and memorize Mr. Zhang's code of 110 rules, including one requiring employees to “love Broad Group.” Another orders them to brush their teeth twice day.

Mr. Zhang makes no apologies for his uncompromising corporate culture.

“This is my personality,” he says.

Yet in the multifaceted Chinese culture another layer might operate. There is today a series of famous Chinese CEOs who have surrounded themselves with formidable top management teams, like Mr. Jack Ma from the e-commerce giant Alibaba and Mr. Zong Qinghou, the billionaire founder of the beverage giant Wahaha. Analyzing Chinese structures is a challenge, starting with the high-context language and its rich repertoire for addressing hierarchy (Fei et al., 1992). Some linguists even argue that Chinese does not possess words for “yes” or “no” in the unequivocal sense that Indo-European languages do (Harbsmeier, 2007). On the other hand, the Janus-faced hierarchical and benevolent leadership legitimate to Confucian followers (Chan et al., 2013) might have led observers to derive “Chinese Teams Like Western Teams (Myth 3).” Of course, the presence of similar visible behavioral phenomena during team functioning (e.g., open discussion) in China and in the West does not imply equal team functioning, especially as the contextual variables mediating the communication process are essentially distinct (e.g., open discussion encouraged by a benevolent leader). To recapitulate, Chinese “imperial courts” may be teams in the sense that they are synergistic and the whole is greater than the sum of the parts or individual members. But they are teams, and high-performing teams, in an indigenous Chinese manner and not in an essentialist Western way.

There is a second and more radical authoritarian tradition in China which is often obviated: Legalism (法家). Legalism, a leading school of thought whose main exponents are the *Han Fei Zi* (韓非子), the summary treatise by the political philosopher Han Fei, and the Book of Lord Shang, the *Shang Jun Shu* (商君書) by the political reformer Shang Yang (商鞅), and which was forcefully implemented in China by the first emperor Qin Shi Huang. The philosophy revolves around strict rewards and punishments and corresponds to an extreme command-and-control approach to leadership (Peng et al., 2008; Feng, 2015; Ma and Tsui, 2015). Legalism is all about rules, management by a bureaucracy that is impersonal and *a priori* just: “The ruler must have clear standards and correct exemplars, as though letting the scales hang to weigh light and heavy, as the means to unify the team of ministers” (Shen Pu-hai 3 in Graham, 1989). Rules also apply to the leader, but rules are different at every hierarchical level. Yet in contrast to Confucianism with its emphasis on loyalty to relationships (Ma and Tsui, 2015), Legalist leadership is assessed as rational, transparent and modern with its sharp focus on merit and performance orientation.

Just like Confucianism requires the effective leader to ultimately work, legalism hinges on effective rules. The leader-follower relationship refereed by rules, the “two handles” of reward and punishment, rather than by benevolence or the pursuit of harmony, implying a deep professionalism, performance-orientation, and the proscription of personal feelings (Witzel, 2012; Lin et al., 2018). Legalism has been associated the with modern transactional leadership theory (Ma and Tsui, 2015; Lin et al., 2018) of Burns where “leaders approach followers with an eye to exchange one thing for another” (Burns, 1978, p. 4). Weber's legal-rational authority and its abstract principles (Clegg, 2015) are a fundament of transactional leadership with its focus on the follower's economic calculus, self-interest and impersonal exchange. Legalism leads to self-management, with action being “outsourced” to a super-structure of strict rules. All in all, Legalist ideas are found to be “highly consistent with the Western Weberian rational model of management and leadership” by Ma and Tsui (2015, p. 19). The constraints associated with fair reward and punishment are not just the means to an end but adherence (by followers) to the rules is the end itself, explaining why the leader might actually withdraw once the rules are institutionalized (Lin et al., 2018, p. 304). Paradoxically this is a state of absent leadership not unlike that found in Daoist invisible rulers.

The opposite of these vertical authoritarian leadership models is found in the Chinese thought system of Daoism, in works like the succinct *Dao De Jing* (道德经), the “Classic of the Way and of Potency” (Feng, 2015: orig. 1948) purportedly written by Lao Zi (老子) or the much longer (80,000 vs. 5,000 characters) work of Zhuang Zi (庄子) named after its author. The *Dao De Jing* is a guide to management or the art of ruling (Graham, 1989), which is an apt contradiction in itself. That is not just because Daoism thrives on paradox and reversal but because, unlike Confucianism or Legalism, it makes no claim to define the right rules or to prescribe conduct. Daoism's notions are not alien to the West; François Quesnay's laissez-faire and Adam Smith's “invisible hand” actually parallel some of its main tenets such as *wei wu wei* (为无为) or “practicing active non-action,” the absence of rules (Barbalet, 2011). Its core argument is *zi ran* (自然) or adherence to nature's self-organizing principles, the proverbial self-balancing Yin-Yang that sees all elements in the world balance endogenously without recourse to external authority (Li, 2012). It is anti-authoritarian in its emphasis on the interconnectedness of all things and in its vision of the universe as dynamic an ever-unfolding emergent state (Feng, 2015).

Daoism's self-governance and related harmony between humans and the elements of nature (the universe, its systems) extends to—most importantly for the understanding teams—the relationships among humans themselves (Prastacos et al., 2012). Relevant Western leadership models for Chinese leaders informed by Daoist philosophy include laissez-faire leadership, servant leadership, authentic leadership, empowering leadership, or paradoxical leadership (Ma and Tsui, 2015, p. 14). The latter style gains relevance under VUCA (volatile, ambiguous, complex, and uncertain) circumstances, which characterize today's business environment and are associated with innovation. Daoism familiarity with paradox enables audacious thinking by embracing “multiplicity, diversity and inter-penetrability” (Luo and Zheng, 2016), such approaches being ideally suited to address ambiguity, complexity, and uncertainty challenges (Lin et al., 2018). Testable hypotheses would probe ambiguity tolerance by management teams on the basis on their cultural heritage.

Daoist leadership might seem “soft” since water is its metaphor of choice. But while apparently unseen, water “is persistent and powerful” thus possessing the faculty to shape surroundings (Li and Cui, 2018, p. 303). Or as Haier's Chairman Mr. Zhang Ruimin directly citing the *Dao De Jing* (verse 78): “There is nothing in the world as soft and weak as water, and yet the firmest and strongest cannot stand up to it” (Chen, 2016). Daoist team leadership conceptualization (water flows to the bottom) is distinct from Legalist or Confucian with top-down (and more or less benevolent or consistent) action on subordinates. Daoism is bottom-up, distributed leadership that aims at high-performance—paradoxically—by living out active non-action, *wu wei* (无为):

“When the Master governs, the people are hardly aware that he exists… The Master doesn't talk, he acts. When his work is done, the people say, ‘Amazing, we did it, all by ourselves.”’ *Dao De Jing* 17 (Ames and Hall, 2010)

The paradoxical pair in the citation (“non-action” that is “active”) points not just at theoretical reconciliation, but also to advancement in applied realms. Daoism has allowed management scholars to develop theoretical-practical frameworks of great originality. Li's Yin-Yang balance (Li, 2012, p. 885–886) proposes an open coexistence between “either/or” and “both/and” for a lasting “either/or” which is patently distinct from the mechanistic and reductionist, contradiction-eliminating Aristotelian logic, or Hegelian dialectics resolving temporary contractions with high-level solutions. Singapore Airlines is a case which can be framed by yin-yang balance where two opposites become a duality that resolves an actual business paradox (one which causes essentialist management frameworks to stumble). The airline achieved “outstanding performance” by means of “effectively implementing a dual strategy: differentiation through service excellence and innovation, together with simultaneous cost leadership”—noteworthy is that a dual “strategy was deemed unachievable by Porter (1985) who held that differentiation and cost leadership must be mutually exclusive” (Heracleous and Wirtz, 2009, p. 274).

Finally, we must conclude our review with Buddhism, which despite its “Western” origin (in India), and in part due to a critical role filling a metaphysical system gap in China, has been considered by various scholars as one of China's main systems of thought of Li (1998). Yet in view of Buddhism's *sui generis* adaption, selection, independent intellectual development and its integration into the Chinese worldview and into Daoism, even to the extent of the emergence of the polemic *huahu* (化胡) theory which saw Buddha a manifestation of Laozi (Zürcher, 2007), we refrain from considering Buddhism as a fourth principal and discrete form of Chinese of thought applicable to teams in our analysis, in consistency with previous positions (Ma and Tsui, 2015; Lin et al., 2018).

### Indigenous Leadership Styles to Teams

One may see Daoism, Confucianism, and Legalism not only as systems of thought but as cognitive contexts that mediate team processes and emergent states of teams. This conceptualization ought to be empirically assessable and is summarized in Figure 1 at the end of this section. As we explore such mediated functioning of Chinese teams, we illustrate our conceptual ideas via a brief selection of cases selected for representativeness from the business press, in accordance to Ma and Tsui (2015).

If the “Chinese imperial court” metaphor is conceived as Confucian, the benign ruler, *renzhu* (仁主), is a leader who manages the team like a father his children: With tough love (爱之深责之切). As in transformational leadership, it motivates subordinates “by winning their hearts” (Confucius, 2009), instead of just relying on punishments as in Legalism. Benevolence has been seen as legitimatizing leadership and extracting the best performance levels from team members (Wu et al., 2012). The absence of the virtue of benevolence (sometimes achieved by feigning its existence) leads to tyranny, fear, and a team that is paralyzed in the face of unpredictable punishment by an emotional leader lacking empathy (多做多错, 少做少错, 不做不错). Benevolence's absence may even lead to rebellion and, in Confucian terms, to the inability to hold onto the team for long, as the legitimizing “Mandate of Heaven,” the proverbial *tian ming* (天命), is lost. As an alternative to Confucianism, the strict, unemotional, potentially efficient and fair version of Legalism does work. It is effective, not through the personal tyranny (暴君) and discretionary rules and favoritism found in some forms of Confucianism, but by means of strict but just processes that supersede benevolence and affective dissonance and transparently reward merit.

A public example of awkward leadership at a Chinese “imperial court” could be seen in the case of Ms. Dong Ming Zhu, CEO of Gree Electric Appliances Inc., who became the laughingstock of Chinese websites in 2015 when she decided that Gree should start making mobile phones. Upon being switched on, the phones greeted the user with a welcoming picture of Ms. Dong herself, but contained old technology and had no clear strategic positioning, the product being neither inexpensive nor high-end. The process ended with her replacement as Chairwoman, but not as CEO (ChinaITNews, 2016; Ho, 2016; WaoNews, 2018). Commentators saw a tyrannical “empress”-decision, enforced through a lack of critical counterarguments from her TMT. It is hard to tell whether she acted as a Confucian or a Legalist leader, as benevolence and punishments can merge in China's authoritarian leadership style.

The potentially disturbing interference of authority is explicitly described in Daoist sources, such as in verse 57 of the *Dao De Jing* (Ames and Hall, 2010): “The more laws and edicts are proclaimed/The more thieves and bandits there will be.” And yet “If I do nothing, of themselves the people are transformed.” At the same time, a functioning self-organizing Daoist *wu wei* organization risks falling into free-for-all chaos without a system that supports bottom-up management, as is the case of the budget hotel chain 7 Days Inn (Qin et al., 2015). This hotel chain has seen unprecedented growth, and is known for its unorthodox hotel management, which has been termed “free-range management.” The board and the CEO cite a well-known Chinese proverb saying that, when the boss has a policy, the employees develop a counter policy (上有政策, 下有对策). To avoid such counterproductive team processes, the CEO cites the *Dao De Jing*, emphasizing that people at HQs (the top Confucian echelons) are not smarter than the local business agents and must not stand in their way. The *wu wei* system of “free-range management” is the brainchild of CEO Mr. Zheng Nanyan; self-organization combines with strong leadership. More generally, and even if seldom in purist Daoist form, self-governing organizations, leaderless networks, ecosystems, are amply documented in China at all levels. For example, the groups of city vendors and tourism service providers who share knowledge and resources in largely self-governing ways (Wen et al., 2016), or Haier's logistics network boasts 90,000 independent drivers (Hamel and Zanini, 2018).

Confident Daoists would point out that team members in a Confucian “imperial court” lack the spontaneity and creativity associated with self-managed ways. The exhortation to the leader in *Dao De Jing* 29 is clear: “Trying to control the world? I see you won't succeed… Those who control, fail. Those who grasp, lose” (Ames and Hall, 2010). Yet Confucian action-oriented “imperial courts” with indisposed top-down vertical hierarchies, may actually degrade into skeptical or masterless (*wu zhu*) organizations. Such a state is not too different from a type of under-performance associated with the chaos or actionlessness, *bu zuowei* (不作为), of failed Daoism. Daoist teams can be dysfunctional too, since without the right team spirit and genuinely shared horizontal leadership, a leaderless team will stall or become mired in the personal ambitions of individual team members. The original productive, competitive and self-managed dynamic of Daoist teams decays into a chaotic state, akin to Confucian teams that have become masterless.

Among the most impressive global Chinese firms, we find Daoist self-governance, like in e-commerce platforms. Alibaba, China's largest e-commerce platform, supplies data and other resources (such enablement would be Confucian) to literally millions of micro firms, entrepreneurs and large firms which then are engaged in commerce in a relatively self-organizing manner. This marketplace model contrasts with the tightly managed and perfectly optimized main pipeline model of its global rival, Amazon, which, one could say, adheres to the Legalist approach. Large Chinese traditional manufacturers can also display Daoist-inspired forms of organization. The big household appliance company Haier announced that it would flatten management hierarchies and remove middle management where possible. Haier's lean, flexible, and customer-oriented organization (Fischer et al., 2013), suggests *wu wei* as does the Haier Open Partnership Ecosystem (HOPE), where “a sea of entrepreneurs” aim at “management without leadership and organization without borders” (Chen, 2016). CEO of Haier, Mr. Zhang Ruimin explicitly draws on Daoism (e.g., even the lowliest employee is “a leader of future business”) to manage innovation (Chen, 2016).

In sum, one might conceive of Chinese teams with the aid of the “imperial court” metaphor and benefiting from three distinct, apparently contradictory, leadership styles anchored in Chinese thought, as seen in Figure 1.

The conceptual development proposed in this paper means that the Chinese “imperial court,” while Confucian, often combines and dearly possess elements of rule-oriented Legalism and self-organizing Daoism along the continuum from ideal to non-ideal team, which manifests itself in practice in more paradoxical and mixed ways than at Western teams. Daoist Haier also implemented a Legalist “Overall Control and Clear” (OCC) management system to “ensure that every employee finishes on his or her job every day, in order to ‘accomplish what’s planned each day and improve on what's accomplished the previous day”' (Chen, 2016). Mr. Ren Zhengfei attributes Huawei's success to following the *zhong yong* (中庸) the doctrine of the (golden) mean (which on account of its balancing quality appears Daoist, but is the title of one the four Confucian classic books). Likewise, Huawei's generous and extensive ESOP (employee stock ownership plan) is seen by the firm's founder as promoting the “Confucian values of equality and harmony,” even as Mr. Ren Zhenfei himself returns to Legalist meritocratic thinking when he criticizes the decision of Mr. Dan Price, CEO of Gravity Payments, to raise the salaries of all employees to US$ 70,000 as unfair and demoralizing to the better producers (Tao et al., 2018). The yin-yang balance framework's “duality” (Li, 2012) contemplates the mixing of all three indigenous leadership styles weighting in with specific teams at different aspects, times and degrees.

Similarly, team processes and other team emergent states are dynamic, just like performance outcomes (Mathieu et al., 2008; Coultas et al., 2014); hence high performance might turn into low and vice versa. This corresponds to the IMOI theoretical understanding of teams undergoing a sequence of performance episodes where, “Outcomes from initial episodes often become inputs for the next cycle. Processes are likely to vary in importance across episodes” (Marks et al., 2001, p. 360; Ilgen et al., 2005). Process theory has recently also been extended, for instance, to explain CEO succession effects in Western companies (Hollenbeck et al., 2015). In China, the three leadership styles at “imperial courts” mediate processes which can lead to a variety of outcomes (performance), as represented in Figure 1. It is an empirical question to settle which leadership styles are most prevalent, and how these mediate processes (such as communications, conflict management, and objective setting) at high-performing Chinese teams.

## Conclusion

This paper's exploration progressed along two lines. First, we wanted to highlight a gap in research on teamwork at Chinese organizations, which is quite salient in top management team literature (Li and Cui, 2018). This situation probably represents one-sided exploitation of Western models in a Chinese environment (Li, 2012). We choose to delineate this shortcoming by describing a three-team-myths structure facilitated by theoretical issues. To that end we compared the well-known team myth in Western contexts “Ideal Team (Myth 1)” with the tacit myth that there are no teams as such in the Chinese context “Chinese Non-Team (Myth 2).” The accumulation of research refuting “Chinese Non-Team (Myth 2)” gave rise to the “Chinese Teams Like Western Teams (Myth 3).” This would be an anti-myth created by the essentialist theory of management team that leaves out Chinese sources of thought as contextual determinant and mediators. Secondly, we strove to replace the (anti-)myth “Chinese Teams Like Western Teams (Myth 3)” by conceptualizing Chinese teams, including invoking the loaded metaphor of an “imperial court.” We proposed that Chinese systems of thought mediate how the team functions in China. Teams mediated by indigenous leadership styles capture the unique nature of Chinese teams and so deny the essentialist “Chinese Teams Like Western Teams (Myth 3),” as we tried to illustrate through a few contemporary cases from Chinese business.

The insufficient conceptualization in mainstream team theory of cultural mediators might wrong practitioners. In this sense, we want to emphasize the usefulness of the myths as heuristic guidelines for future research. The conceptual development around how Chinese systems of thought impact Chinese team processes is falsifiable and accessible to empirical testing. Confucian, Daoist, and Legalist leadership styles and their mediating role in team processes and organizational behaviors can be conceptualized, coded, and tapped in observation of practice, as for example done with Daoist thought in a Western setting (Manz and Sims, 2001) or with the influence of Mao Zedong thought (Lin and Clair, 2007).

Beyond the present essentialist and ethnocentric constraints on research awaits a rich empirical research agenda. We suggest the tighter, maximalist indigenous research position beyond mere contextualization as we aim to develop indigenously derived theory (Tsui, 2004; Li, 2012) for general knowledge and universal adaptation (Li, 1998).

A better understanding of the mediating effects of cultural context manifested in Chinese leadership styles, on team processes, emerging states, and outcomes arguably invigorates the development of general management team theory. Just as Western theories have been imported to Chinese management for some years, the reverse is equally productive. We hinted at this possibility earlier when we compared Amazon with Alibaba and framed their respective models in Daoist and Legalist terms. So did Hamel and Zanini's (2018) call for “The End of Bureaucracy” referencing Haier, where vigorous employee-entrepreneurs organized into 3,800 “microenterprises” productively interact in an open self-managed ecosystem with partners, customers and inventors, all in lieu of formal hierarchy. Ancient Chinese proverbs have been included in Western management texts in many instances over the years, but usually only as illustrating aphorisms. There are some promising exceptions. For example, Charles Manz has built a systematic theory of self-leadership, servant leadership and self-governing teams building explicitly on the Dao De Jing (Manz and Sims, 2001).

Manz self-leadership theory is liable to falsification. Falsifiable hypotheses resulting from this contribution on Chinese TMTs would want to ambitiously focus on firm performance as the outcome variable in the IPO and IMOI tradition. The input-output template would emphasize the mediator roles of, for instance interactions associated with determined systems of thought. The operationalization of the system of thought, which is necessary for testing, would be carried out though concreate observable phenomena that is sufficiently distinct form the West, as is the “paternalistic care of employees.” A company ran by a Chinese-style Confucian “court” in our terminology, would be assessed in terms of hard behavioral evidence to be able to claim benevolent paternalistic ruler (Chan et al., 2013). Thereafter the “imperial court” teams would be tested for their synergies in terms of high-performance, but on account of the previously noted operationalization in an indigenous Chinese manner and not in an essentialist Western way. Likewise a paradoxical leader (Zhang et al., 2015b) would be evidenced by the implementation in the organization of open but competitive initiatives internally—Daoist style, as in 7 Days Inn (Qin et al., 2015) or to some extent in Alibaba (Ma, 2013; Ming, 2018) self-governing e-commerce platforms.

In short testable hypothesis would see the operationalization of cultural discrete behaviors and phenomena associated with an indigenous leadership style. We have suggested a series of falsifiable hypotheses throughout this contribution and more could be built around Chinese companies applying, for instance, the operationalized properties and communication interactions of an “imperial court” or “action-less” leadership to foreign subsidiaries. The essential insight is that Chinese heritage and conceptualization such as active non-action teams or benevolent leaders at “imperial courts” represent a vast management know-how treasure trove. It would be a disservice to team research if it were held down by essentialist conceptions and misconceptions of teams. Instead, a richer indigenous perspective helps the academy theorize and understand Chinese and *all* teams. Practitioners also need broader perspectives, in cross-cultural contexts and beyond, related to the ultimate dependent variable of teamwork—performance.

## Author Contributions

TC and JA searched and reviewed existing literature, co-operated in creating the conceptual models, integrated real-life business cases, and co-wrote the manuscript. All authors contributed to the article and approved the submitted version.

## Conflict of Interest

The authors declare that the research was conducted in the absence of any commercial or financial relationships that could be construed as a potential conflict of interest.

## Footnotes

^1^https://en.oxforddictionaries.com/english
